# Distribution of Cardiac Stem Cells in the Human Heart

**DOI:** 10.5402/2012/483407

**Published:** 2012-03-04

**Authors:** Mani Arsalan, Felix Woitek, Volker Adams, Axel Linke, Markus J. Barten, Stefan Dhein, Thomas Walther, Friedrich-Wilhelm Mohr, Jens Garbade

**Affiliations:** ^1^Department of Cardiac Surgery, Kerckhoff Klinik, Bad Nauheim, Benekestr. 2-8, 61231 Bad Nauheim,, Germany; ^2^Department of Cardiology, Heart Center Leipzig, University of Leipzig, Struempellstrasse 39, 04289 Leipzig, Germany; ^3^Department of Cardiac Surgery, Heart Center Leipzig, University of Leipzig, Struempellstrasse 39, 04289 Leipzig, Germany

## Abstract

*Introduction*. The existence of human cardiac stem cells (hCSC) and their regenerative capacity are not fully defined. The aim of this study was to identify and analyse the distribution of hCSCs by flow cytometry (FCM). *Methods*. Tissue samples from the left ventricle (LV) and the appendages of the right atrium (RA) and left atrium (LA) were taken during cardiac surgery. Mononuclear cells (MNCs) were isolated, labelled for the stem-cell-marker c-kit and hematopoietic-lineage markers and analysed by FCM. *Results*. HCSCs could be isolated from the RA, LA, and LV without significant quantitative difference between both atria (A) (RA 4.80 ± 1.76% versus LA 4.99 ± 1.69% of isolated MNCs, *P* = 0.922). The number of hCSCs was significantly higher in both atria compared to the left ventricle (A 4.90 ± 1.29% versus LV 0.62 ± 0.14% of isolated MNCs, *P* = 0.035). *Conclusion*. The atria contain a higher concentration of hCSC than the left ventricle. HCSCs located in the atria could serve as an endogenous source for heart regeneration.

## 1. Introduction

Despite various treatment options, heart failure is still the leading cause for mortality and morbidity in the elderly. In the last years stem cell transplantation for the purpose of cardiac regeneration was successful in experimental studies.

Diverse pluripotent endogenous adult stem cells were tested for their impact on myocardial regeneration [1–4]. Clinical trials focussed on bone-marrow-derived stem cells to initiate cardiac regeneration and showed an improvement of cardiac function [5]. Nevertheless, the search for more applicable cells with a better outcome still continues.

The human heart has always been defined as a postmitotic organ with a determined number of cardiomyocytes (CMs) formed during the embryonic and foetal life. Thus, it was assumed that if the heart loses a number of CMs, the remaining cells would have to sustain the heart function.

The identification of human cardiac stem cells (hCSC) revealed the heart's own capacity for regeneration. Furthermore, it was reported that cell turnover occurs in the human heart [1]. This suggests that the CMs undergo apoptosis at a certain rate and are regenerated by hCSCs.

The existence of hCSC was reported by several researchers, but their origin, function, and possible therapeutic benefit are still under discussion [6].

The cardiac distribution of hCSCs in patients with heart diseases, a basic requirement for their therapeutic use in the future, is not yet determined.

Therefore, the aim of the present study was to investigate the distribution of hCSC in different compartments of the heart with the help of flow cytometry.

## 2. Materials and Methods

Myocardial tissue samples (*n* = 20) were taken from the left ventricle (LV), the appendages of the right atrium (RA) and left atrium (LA) from adult patients undergoing cardiac surgery. The average age of the patients was  67 ± 2  years. The samples were taken during aortic valve replacement, mitral valve repair/replacement, and coronary artery bypass surgery. The samples weight was 0.36 ± 0.09 g.

To confirm the FCM results, several tissue samples were additionally analysed by immunohistochemistry. This study was approved by the local ethical committee and followed the rules of the Helsinki Declaration for patient dates and evaluation. Informed consent was given by the patients.

### 2.1. Flow Cytometry

The tissue samples were weighed and washed several times in Hank's Balanced Salt Solution, followed by a sequential digestion with collagenase IV and trypsin (15 min, 37°C, 0.2 mg/mL). The cell suspensions were filtered using cell strainer (100 *μ*m, 70 *μ*m, and 40 *μ*m) and MNCs were isolated by density gradient centrifugation. These cells were stained with specific antibodies (100 *μ*L cells suspension + 5 *μ*L of each antibody incubated for 20 min.) for stem cell marker c-kit (Polyclonal rabbit Anti-Human CD 117, Dako) and the hematopoietic lineage markers CD3, CD11b, CD19, and CD45 (antihuman, BD Biosciences). The nuclei of the cells were labelled with draq 5 (Biostatus, 0.5 *μ*L was added 10 min. after the other antibodies). Cell characteristics were analysed using a LSR II flow cytometer (BD Biosciences, San Jose, CA).

### 2.2. Immunohistochemistry

The tissue samples were fixed in 4% Phosphate buffered saline buffered formalin and embedded in paraffin.

For immunofluorescence staining, the sections were deparaffinized in xylene, rehydrated in alcohol series (1 × 10  minutes 100%, 1 × 10 minutes 96%, and 1 × 10 minutes 76%), dried for 10 minutes, rehydrated for 5 minutes in distilled water, and washed in Tris Buffered Saline (TBS) for 10 minutes. Antigen retrieval was performed by boiling the section in Na-Citrate (10 mmol/L) for 30 min. using a microwave (30 min. at 800 Watt). The sections were cooled down for 30 min., before they were washed in TBS for 10 minutes and blocked with 4% milk/TBS for 1 h at room temperature. Subsequently, the sections were incubated with the primary antibody (Polyclonal rabbit antihuman CD 117, c-kit, Dako) over night at 4°C. On the following day, the sections were washed three times for 5 minutes in Tris-NaCl-Tween-Buffer (TNT). The sections were blocked with TNB-Buffer (TNT buffer containing blocking reagent) for 30 minutes and incubated with a secondary antibody (Goat-Anti Rabbit, Dianova) for 30 minutes at room temperature. They were washed in TNT-Buffer for 3 × 5 minutes and treated with biotinylated tyramid for 10 minutes. The sections were washed in distilled water and mounted with Fluorescent Dako.

Quantitative and qualitative histological analyses were performed using an Axioplan2 microscope (Carl Zeiss GmbH, Jena, Germany) and the KS 300 Imaging System 3.0 (Carl Zeiss Vision GmbH, Eching, Germany).

### 2.3. Statistical Analyses

The multivariate data analysis was performed by FACS Diva software (BD Biosciences, San Jose, CA). All data are expressed as mean and ± SEM. Statistical comparison was performed by one-way ANOVA followed by paired *t*-test as appropriated. Results were considered statistically significant as *P* < 0.05. All data analyses were performed by using SAS software, version 6.11 (SAS Institute, Cary, NC, USA).

## 3. Results

### 3.1. Flow Cytometry

**Figure 1:**
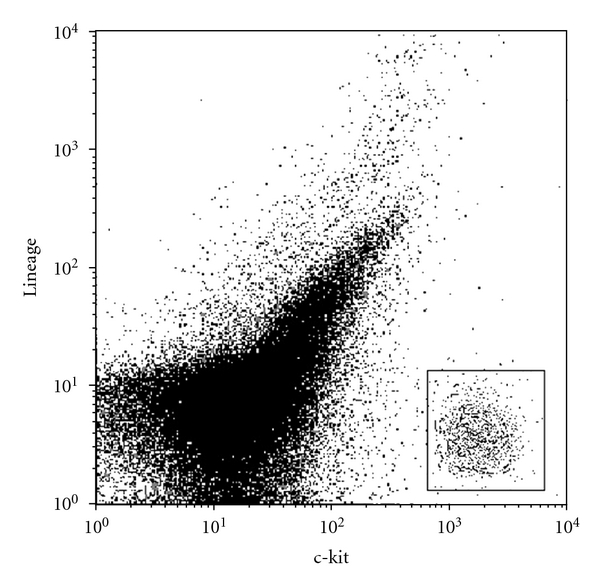
FCM analysis of c-kit/lineage of atrial tissue.

**Figure 2:**
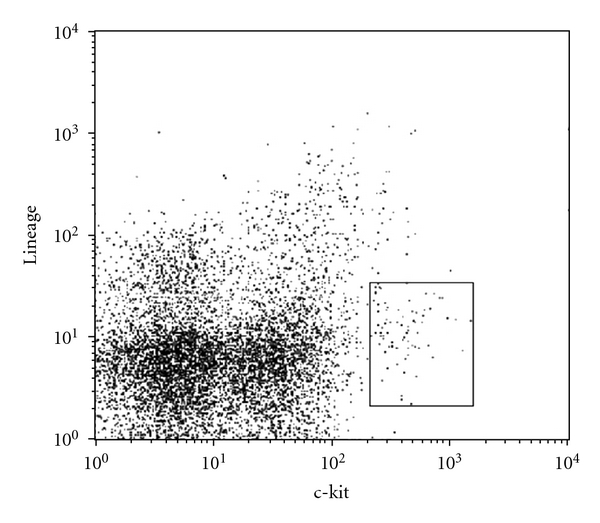
FCM analysis of c-kit/lineage of left ventricular tissue.

With the mentioned approach, we could isolate MNCs from heart tissue and identify c-kit^pos^ cells in all samples. We detected human cardiac stem cells which were c-kit^pos^ and lineage^neg^ in all investigated heart compartments (Figures 1 and 2).

**Figure 3:**
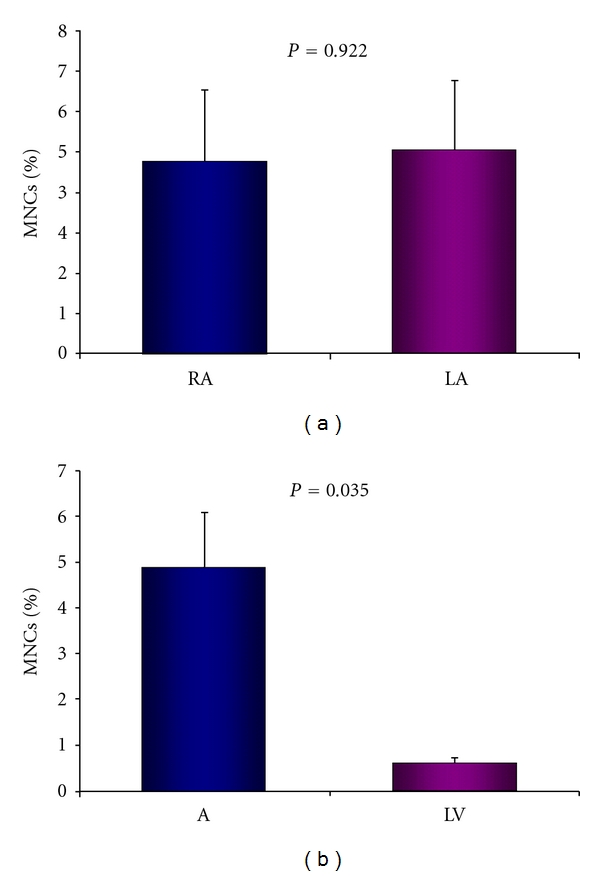
(a) Comparison of c-kit^pos^/lin^neg^ cells between the right (RA) and left atrium (LA), (b) Comparison of c-kit^pos^/lin^neg^ cells between the atria (A) and left ventricle (LV).

There is no significant quantitative difference of c-kit^pos^ and linage^neg^ cells between both atria (A) (RA 4.80 ± 1.76% versus LA 4.99 ± 1.69% of isolated MNCs, *P* = 0.922, Figure 3). The number of c-kit^pos^ and linage^neg^ cells was significantly higher in both atria compared to the left ventricle (A 4.90 ± 1.29% versus LV 0.62 ± 0.14% of isolated MNCs, *P* = 0.035, Figure 3).

### 3.2. Immunohistochemistry

**Figure 4:**
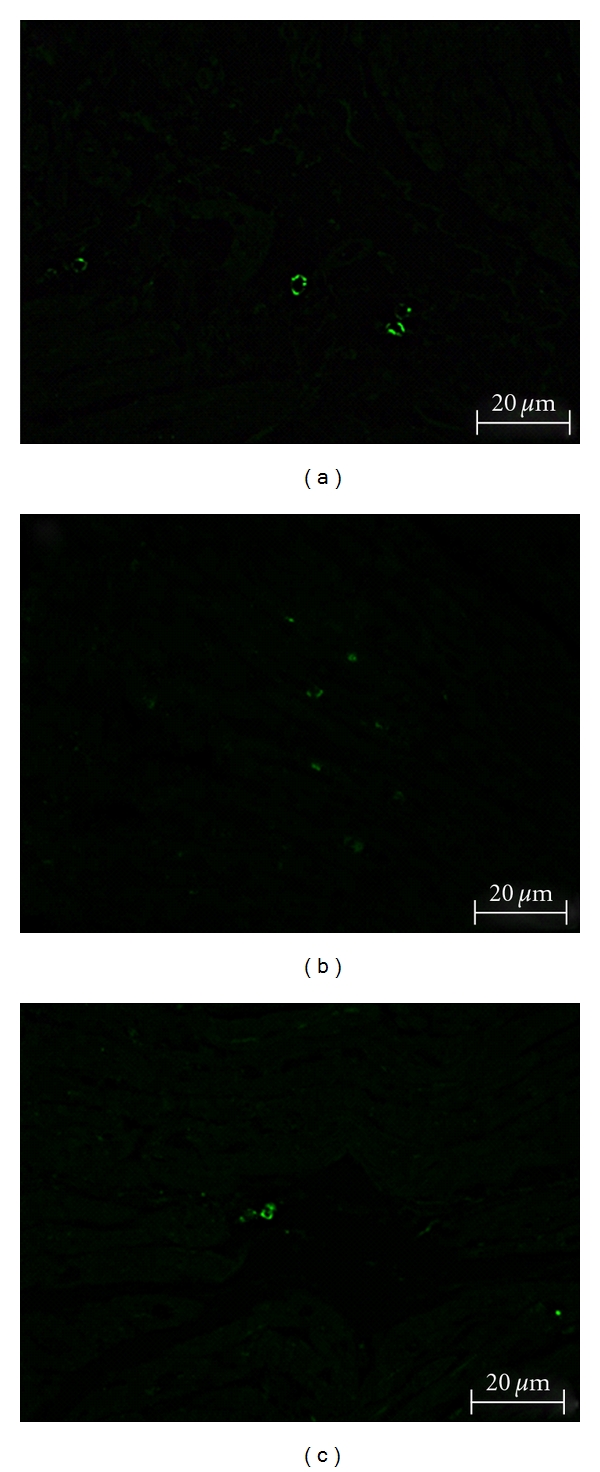
c-kit positive cells embedded in myocardial tissue; (a) left atrium, (b) right atrium, (c) left ventricle.

The immunohistochemical staining showed c-kit^pos^ cells in all investigated heart compartments and confirmed the distribution shown by FCM analysis (Figure 4).

## 4. Discussion

Several reports support the existence of cardiac stem cells in the adult heart, but only a few studies used human tissue samples. In this study, we report the presence and distribution of human cardiac stem cells defined by the expression of the cell surface antigen c-kit and the absence of hematopoietic lineage markers in patients undergoing cardiac surgery.

Our data support other reports about a c-kit-positive population of cardiac stem cells and extend these findings by showing a significant difference in the cell distribution between the atria and the left ventricle.

Many clinical studies investigated the influence of stem cell transplantation on heart function after myocardial infarction or cardiomyopathy. After the initial demonstration of safety, especially bone-marrow-derived stem cells were used in clinical trials to initiate cardiac regeneration [7–12]. Other studies using growth factor or other stimulating factors demonstrated similar effects [13]. Both approaches lead to an improvement in heart function. Whether these effects are due to transdifferentiation into CMs, induction of angiogenesis, or paracrine effects on hCSCs is still under discussion [14]. Maybe all three mechanisms are involved [15].

Current investigations focus on finding the ideal cell type for cell therapy as each one has its own benefits and disadvantages.

Bone-marrow-derived stem cells (BMCs) are easy to gain and their transplantation leads to a light improvement of cardiac function for about 2 years and reduces the occurrence of major adverse cardiovascular events [16, 17]. Lin et al. reported that endothelial progenitor cells (EPCs) derived from bone marrow play an important role in angiogenesis [18]. It could be shown that erythropoietin improves cardiac function by homing and incorporating EPCs into the myocardial microvasculature and myocardial secretion of angiogenic factors [19].

But as EPCs only seem to improve vascularization, regeneration of the heart by creation of new CMs is not expected.

It was reported that skeletal myoblasts can differentiate into viable muscle fibres within the scarred tissue after transplantation [20]. However, in a clinical trial myoblast transfer did not improve LV function compared to placebo, but increased the number of early postoperative arrhythmic events [21].

Ii et al. showed that adipose-derived stem cells also exhibit a therapeutic effect on cardiac preservation following myocardial infarction [22]. This positive effect is not due to transdifferentiation of the cells. One explanation may be the production of growth factors like VEGF, bFGF, and SDF-1*α* showing paracrine effects by supporting endogenous progenitor cell recruitment to ischemic myocardium [22]. Another study by Gaebel et al. showed that bone-marrow-derived human mesenchymal stem cells initiate a greater cardiac improvement in comparison to those from adipose tissue [23].

Cardiac stem cells represent a promising source for cell therapy as they seem to be the physiological depot for cardiac regeneration. A high regenerative potency and low risk for arrhythmias are assumed.

If the hCSCs origin is really in the myocard or if these cells are provided by the bone marrow is not clear yet, but at least a part of hCSCs seem to have their origin in the bone marrow [15]. Regeneration implies that dead cells are replaced by newly formed cells restoring the original structure of the organ. It was shown that hCSCs can differentiate in vitro and in vivo to myocyte, smooth muscle and endothelial cell lineages [24].

In adulthood, this occurs during physiological cell turnover, but myocardial damage could stimulate the differentiation of resident hCSCs into de novo cardiomyocytes. Mishra et al. recently reported that hCSCs are abundant in the neonatal period and decrease over time [25]. Our observed differences in distribution support this hypothesis as the transdifferentiation of hCSCs would primarily occur in the ventricle where a loss of CMs is more likely. The reduced amount of hCSCs explains the hearts inability to regenerate in the elderly and could be the reason why the benefit of stem cell transplantation is limited.

Consequently, increasing the number of hCSCs may boost the regenerative capability of the heart. As several reports showed an improvement of heart function after the injection of hCSCs in the heart, a therapeutic approach could be to isolate hCSCs, expand them in vitro, and transplant them back to the same patient [26–28].

Another option could be the injection of substances leading to a migration and/or proliferation of CSCs. Linke et al. and Rota et al. reported that the activation of resident CSCs by hepatocyte growth factor and insulin-like growth factor-1 as well as the injection of CSCs in the heart leads to de novo myocytes and vascular structures [13, 27].

Tang et al. reported that the injection of exogenous CSCs activates endogenous CSCs and is beneficial in the setting of an old myocardial infarction [29].

Additionally, the positive effects on contractile behaviour seem to be independent of the CSC donors age. Thus, CSCs could be the ideal cell for cardiac regeneration [30].

## 5. Conclusion

Cell therapy is a promising strategy to treat heart failure, as it aims to regenerate the myocardium with contractile substance. Up to now, the ideal cell type is still unknown. Since the discovery of CSCs, researchers investigate different ways of using these cells for cardiac regeneration. As far as we know, this is the first report about the distribution of hCSC in the different compartments of the heart. We show that the concentration of CSCs is higher in the atria than in the ventricle. This suggests the use of the atria as the origin for CSC gaining. As myocardial infarctions usually hit the ventricle, the atria could serve as a source for cardiac regeneration. Therefore, the arrhythmogenic impact and potential for differentiation of these cells should be investigated.
